# Transport Rankings of Non-Steroidal Antiinflammatory Drugs across Blood-Brain Barrier In Vitro Models

**DOI:** 10.1371/journal.pone.0086806

**Published:** 2014-01-23

**Authors:** Iveta Novakova, Eva-Anne Subileau, Stefan Toegel, Daniela Gruber, Bodo Lachmann, Ernst Urban, Christophe Chesne, Christian R. Noe, Winfried Neuhaus

**Affiliations:** 1 Department of Medicinal Chemistry, University of Vienna, Vienna, Austria; 2 Biopredic International, Rennes, France; 3 Department of Orthopedics, Medical University Vienna, Vienna, Austria; 4 Core Facility Cell Imaging and Ultrastructure Research, University of Vienna, Vienna, Austria; 5 Department of Anesthesia and Critical Care, University Hospital Würzburg, Würzburg, Germany; Oregon Health & Science University, United States of America

## Abstract

The aim of this work was to conduct a comprehensive study about the transport properties of NSAIDs across the blood-brain barrier (BBB) in vitro. Transport studies with celecoxib, diclofenac, ibuprofen, meloxicam, piroxicam and tenoxicam were accomplished across Transwell models based on cell line PBMEC/C1-2, ECV304 or primary rat brain endothelial cells. Single as well as group substance studies were carried out. In group studies substance group compositions, transport medium and serum content were varied, transport inhibitors verapamil and probenecid were added. Resulted permeability coefficients were compared and normalized to internal standards diazepam and carboxyfluorescein. Transport rankings of NSAIDs across each model were obtained. Single substance studies showed similar rankings as corresponding group studies across PBMEC/C1-2 or ECV304 cell layers. Serum content, glioma conditioned medium and inhibitors probenecid and verapamil influenced resulted permeability significantly. Basic differences of transport properties of the investigated NSAIDs were similar comparing all three in vitro BBB models. Different substance combinations in the group studies and addition of probenecid and verapamil suggested that transporter proteins are involved in the transport of every tested NSAID. Results especially underlined the importance of same experimental conditions (transport medium, serum content, species origin, cell line) for proper data comparison.

## Introduction

The blood-brain barrier (BBB) maintains the homeostasis between blood circulation and the central nervous system (CNS). It consists of brain microvascular endothelial cells with distinct different features in comparison to the peripheral endothelium. Major brain endothelium specific properties are the lack of fenestrae, reduced endocytosis and restricted paracellular transport [1]. The barrier functionality comprises a physical, a transporter and a metabolic component. Physical tightness of the barrier is determined by tight junction proteins such as occludin, claudin-3 or claudin-5 which seal the paracellular gaps and consequently restrict the permeation of hydrophilic compounds. Transcellular migration could be regulated by influx as well as efflux transporter proteins. Lipophilic substances could permeate by passive diffusion across the cell membranes or by being shuttled via transporter proteins. Hydrophilic molecules such as glucose need transporters such as glut1 to overcome the BBB and reach the CNS. In addition to defend against pathogens such as viruses or bacteria the BBB can also recognize substances and actively efflux them back into the bloodstream. ATP-binding cassette (ABC)-transporters such as ABCB1 (P-gp), ABCG2 (Bcrp) or ABCCs (multidrug resistance related proteins  =  MRPs) play a major role in these protection mechanisms. As third component a huge array of enzymes can metabolize substances and prevent their CNS entrance by molecular conversion and/or conjugation. Barrier functionality is regulated by the microenvironment of the capillary endothelium. The terms neuro/gliavascular unit describe that astrocytes, pericytes and neurons can interact and modify endothelial functional properties. In addition, shear stress by the bloodstream applied onto endothelial cells was shown to tighten the barrier in vitro [2]–[4].

Alterations of BBB functionality during several diseases such as Alzheimer’s disease, Parkinson disease, multiple sclerosis, stroke, traumatic brain injury and many more have been observed [5]–[9]. Inflammation is an important component in disease progression of some of these diseases which could be treated by administration of non-steroidal anti-inflammatory drugs (NSAIDs) [10]. For example, application of ibuprofen was shown to reduce the risk to suffer from Alzheimer’s disease [11]. NSAIDs block activity of cyclooxygenases (COX) with different COX1/COX2 inhibition profiles and subsequently reduce the production of prostaglandins, prostacycline and thromboxane A2. In general, NSAIDs reduce fever and pain, stop inflammatory processes and could be used for antiaggregation. In addition to side effects in the periphery such as ulcerates, erosion in digestive tract, nausea, gastritis, bleeding, diarrhoea or constipation, several central side effects like dizziness, headaches and drowsiness, depressions, hearing and visual impairment, tinnitus, etc. are known [12]–[14]. CNS side effects implies BBB permeability of NSAIDs as prerequisite to reach their place of action. In humans as well as in several animal models it was proved that NSAIDs can cross the BBB [15]–[20]. Nonetheless, no comprehensive, systematic study about the permeability of NSAIDs and their classification with regard to their permeability ranking exist. Consequently, the aim of this study was to investigate the transport of several NSAIDs across the BBB in vitro. Transport of NSAIDs with different COX1/COX2 inhibition profiles (preferentially COX1-inhibition: ibuprofen, piroxicam, tenoxicam; preferentially COX2-inhibition: meloxicam, diclofenac; COX2-inhibition: celecoxib) was studied in three different BBB in vitro models which differ in species origin and barrier properties. Beginning with single substance studies, group studies including several NSAIDs and internal standards within one study should further elucidate the influence of different experimental conditions (serum content, astrocyte factors, group composition, addition of efflux transporter inhibitors verapamil and probenecid) and provided a general view about the transport rankings of the investigated NSAIDs.

## Material and Methods

### Material

Celecoxib, diclofenac, lornoxicam and diazepam were a kind gift of Dr. Maierhofer (AGES, PharmMed, Austria), whereas ibuprofen (I1892, SigmaAldrich, Austria), meloxicam (M3935, SigmaAldrich, Austria), piroxicam (P5654, SigmaAldrich, Austria), tenoxicam (T0909, SigmaAldrich, Austria), carboxyfluorescein (21877, Fluka, Switzerland), probenecid (P8761, SigmaAldrich, Austria) and verapamil (94837, Fluka, Switzerland) were purchased from commercial sources. Iscove’s modified Dulbecco’s medium (IMDM), Ham reg. nutrient mixture F12 (Ham’sF-12), newborn calf serum (NCS), l-glutamine and penicillin/streptomycin were obtained from Invitrogen Life technologies (GibcoTM, Carlsbad, CA). Heparin and collagen solution (predominantly collagen I, 150703) were purchased from MP Biomedicals (Irvine, CA). Amphotericin B, transferrin, 8-aminopyrene-1,3,6-trisulfonate (APTS) and gelatine were from SigmaAldrich (Austria), whereas dextran (av. MW 6000) was from Fluka (Switzerland). Fibronectin was obtained from BD Biosciences (Bedford, MA) as well as Transwell inserts (FalconTM) and six-well plates (FalconTM). Basal endothelial and astrocyte media and components for primary rat endothelial cells and astrocytes were provided from Biopredic Int. (France). Inorganic salts and all other reagents were of analytical grade.

### Cell culture

Porcine cell line PBMEC/C1-2 was a kind gift from Teifel and Friedl, which was established and characterized by them [21]. Human ECV304 cells were purchased from the European Collection of Cell Cultures (ECACC, Wiltshire, UK), rat glioma cell line C6 was obtained from the German Cancer Research Center Heidelberg (DKFZ, Heidelberg, Germany). Cell culture conditions were previously reported [22]–[27]. In brief, C6 cells were cultured in gelatin-coated 175 cm^2^ tissue flasks (Greiner BioOne GmbH, Germany) with so-called C6 medium, which consisted of a 1∶1 mixture of IMDM and Ham’s F-12, 7.5% (v/v) NCS, 7 mM L-glutamine, 5 mg/mL transferrin, 0.5 U/mL heparin, 100 U/mL penicillin, 100 mg/mL streptomycin, and 0.25 mg/mL amphotericin B. Supernatants of C6 cultures were collected every other day and termed glioma-conditioned medium (GCM). PBMEC/C1-2 (passages 73–88) as well as ECV304 (passages 151–174) cells were grown in 25 cm^2^ gelatin-coated tissue culture flasks with so-called PBMEC medium (50% C6 medium, 50% GCM). PBMEC medium was sterile filtered before usage. Primary RBMECs (END105) isolated from 5 weeks old male wistar rats and rat glial cells (AST105) derived from newborn pups were obtained from Biopredic Int. (France) and were cultured according to the manufacturer‘s instructions. Rat astrocytes were grown in gelatin-coated 25 cm^2^ tissue flasks in the medium provided by Biopredic Int. at passages 1–8. RBMECs were defrozen and seeded directly onto 12-well Transwell inserts. All cells were cultured in a humidified 5% CO_2_/96% humidity at 37°C and subcultivated by trypsination every 3 to 4 days.

### Transport studies

6-well Transwell® inserts (1 µm pore size, PVDF) were coated with collagen and fibronectin for PBMEC/C1-2 and with collagen only for ECV304 cells. For permeation experiments cells were seeded at a density of 8*10^4^ cells/cm^2^ and grown to confluency on membrane filter inserts. Culture medium was changed every day (PBMEC/C1-2) and every other day (ECV304) up to the transport studies. For enhanced attachment of PBMEC/C1-2 cells PBMEC medium was supplemented with 1 mg/mL fibronectin. Cell monolayers of PBMEC/C1-2 were used for transport studies on day 3 and layers of ECV304 cells on day 14 after seeding. In case of RBMEC, cells were seeded at a density of 8*10^4^ cells/cm^2^ onto 12-well Transwell® inserts (1 µm pore size, PVDF), which were coated with attachment cell factors 1∶10 diluted CF1 and 1∶100 diluted CF2 (175 µL/insert AFC001003; 150 µL/insert AFC002004) for 1 hours each at 37°C (Biopredic Int., France), in endothelial BBB culture medium (100 mL, MIL121) supplemented with FCS (5 mL, SER037) and additives 1-8 (400 µL MIL045, 100 µL MIL046, 100 µL MIL047, 100 µL MIL048, 40 µL MIL049, 500 µL MIL050004, 50 µL MIL051004, 100 µL MIL052003). Inserts were put in 12-wells containing primary rat glial cells, which had been seeded at a density of 5*10^3^ cells/cm^2^ in glial BBB culture medium (100 mL, MIL043) supplemented with FCS (5 mL, SER036), human AB serum (1 mL, SER035), penicillin/streptomycin 100X (1 mL, PEN016) and additive for glial cell medium (100 µL, MIL044) one day before. Every other day total endothelial medium, but only half of the glial medium was changed until TEER was over 100 Ohm*cm^2^ and co-culture was ready for conduction of the transport study. Transport studies were carried out by transferring inserts at given time points into new wells filled with prewarmed and fresh transport medium as previously published in detail [23]. In case of RBMEC, the basolateral wells were filled with conditioned medium of primary rat glial cells to exclude possible uptake of drugs by glial cells during transport studies. TEER measurement was performed with a Millipore Millicell Electrical Resistance System (ERS, Millipore, Vienna, Austria) after changing the medium and temperature equillibration at room temperature for at least 30 min. To calculate TEER values measured electrical resistances of inserts without cells were subtracted from values with cells and multiplied by the surface area of the inserts (6-well: 4.2 cm^2^, 12-well: 0.9 cm^2^) according to Neuhaus et al. (2006) [22].

Experimental solutions were prepared ‘ad hoc’ from stock solutions. It always contained 100 µM of the respective NSAIDs and 1% DMSO in total. Internal standards were also added (100 µM diazepam, 5 µM carboxyfluorecein (CF) ). All solutions were prepared under sterile conditions. At the end of transport studies, the supernatant of the inserts was removed and analyzed to estimate substance recovery rates. Residual applied stock solution and samples were collected and stored until fluorescence or HPLC analysis at 4°C. Labelling and production of APTS-dextran for the RBMEC study was accomplished according to protocols published recently [22].

### Fluorescence measurements

Fluorescence of carboxyfluorescein was determined by using a microplate reader (polarstar galaxy, BMG Labtech, Offenburg, Germany) at an excitation wave length of 485 nm and an emission filter at 520 nm. Triplicates per sample were measured and blank medium values were substracted. Fluorescence of total APTS-dextran was measured as described above, separation and fluorescence measurement of single APTS-dextran fractions by capillary electrophoresis were carried out as previously published [22].

### Reverse-Phase High-Performance Liquid Chromatography of NSAIDs

For quantification of NSAIDs a reverse-phase high-performance liquid chromatography (RP-HPLC) system from Shimadzu was used (DGV-20A5/prominence Degasser, SIL – 20AC/prominence Auto Sampler, CTO – 20AC/prominence Column Oven, SPD – 20A/prominence UV detector, CMB – 20A/prominence Communications Bus Module). Separation of compounds was carried out using Lichrospher columns from Merck KGaA (Germany, RP-18, 250×4 mm, 5 µm pore size) with LichroCART 4-4 precolumns or Zorbax SB-C8 norrowbore columns (820975-906, 50×2.1 mm, 5 µm pore size, Inula, Austria) with a 2.1×12.5 mm precolumn packed with the same material (821125-915, Inula, Austria). Before analysis proteins of samples were precipitated with ice-cold methanol or acetonitril depending on mobile phase composition and supernatants were analyzed. Data aquisition and analysis were performed by LC-Solution software (Shimadzu Handelsgesellschaft, Austria). Substances were analysed in triplicates with a injection volume of 20–50 µL.Varying mixtures of methanol, acetonitrile, and 10 mM potassium buffer (pH = 2.5 for all analysis except pH = 3.5 for single studies with celecoxib) were used as mobile phase. Analyses were conducted at 25°C, run time was between 8 and 18 minutes with a flow rate of 1 or 1.3 mL/min. Substances were UV-detected at 220 nm (diazepam, diclofenac, ibuprofen and celecoxib in group studies), 254 nm (celecoxib in single studies) or 370 nm (meloxicam, tenoxicam, piroxicam).

### Calculation of permeability coefficients

Permeability coefficients were calculated following the clearance principle as described recently [23]. Results of blank value experiments were included into permeability coefficient calculation by using reciprocal correlation shown in equation (1):

1/*PEcell*  =  1/*PEall* – 1/*PEblank* (1)

PEblank refers to the permeability coefficient without cell layer, PEall represents the permeability coefficient through membrane insert and the cell layer and PEcell is the permeability coefficient only through the cell layer.

### Serum binding measurements

Substance solutions with the same composition for group transport studies in serum-free and C6 medium (100 µM diazepam, 100 µM of each NSAID, 5 µM carboxyfluorescein, 1% DMSO end concentration) were prepared in pure PBS or C6 growth medium supplemented with 0%, 7.5%, 50% or 100% newborn calf serum. After incubation at 37°C for 40 minutes, 2 mL of the solutions were centrifuged across prewashed (2 mL PBS, 10 minutes, 2500 g) Centrisart ultra-filters (molecular cut-off 10 kDa, Sartorius) at 2500 g for 5 (PBS and 0% serum), 10 (7.5% serum) or 15 (50% and 100% serum) minutes to obtain at least one third to one half of the totally applied volume. NSAID concentrations of stock solutions, retentates and filtrates were analyzed by HPLC similar as described above using an Oligo-RP clarity HPLC-column (5 µm pore size, 250×4.6 mm, Phenomenex) at a flow rate of 1 mL/min with a 70:30 eluent mixture of CH_3_CN:potassium phosphate buffer (10 mM, pH = 2.5). Carboxyfluorescein was measured by the microplate reader method as described above. Unbound substance concentrations in the filtrates were related to stock solutions to determine the serum binding (100%-unbound fraction [%]). Blank experiments (PBS, 0% serum experiments) were included in the calculation of serum binding values to consider systematic procedure errors and binding of the substances to the ultrafiltration membrane. Experiments under each condition were repeated for three times.

### RT-qPCR

RT-qPCR of RBMEC lysates was accomplished according to the protocol published previously [24]. Following primer pairs obtained from Metabion were used: ICAM-1 forward: TTCAAGAATGTCTCCGAGGTCAGG, ICAM-1 reverse: TGTTTGTGCTCTCCAGGGTCAG, VCAM-1 forward: ACACAGCAGTCAAATGGAGTC, VCAM-1 reverse: AGCAGGTCAGGTTCACAGG, PECAM-1 forward: GTGCTTCGGTGCTCTGTG, PECAM-1 reverse: ATGCTGGCTCTGTTGAACG, CD44 forward: AACTACAGCCTTGATGACTACC, CD44 reverse: GATGACTCTTGGACTCTGATGG, Claudin-3 forward: CCTTGCTGTGTTGCTCCTG, Claudin-3 reverse: CGGTTGGTAGTGGTGATGG, Claudin-5 forward: GAGCAGAGGCACCAGAATC, Claudin-5 reverse: CAGACACAGCACCAGACC, Claudin-12 forward: CTGCGACTCATCACATTCAAC, Claudin-12 reverse: GTCACTGCTTCCGTCATACC, Occludin forward: TTGTATAAGTCACCGCCTCTG, Occludin reverse: TCTGTCCTCTTCGCCTTCC, ZO-1 forward: GCCAAGCCAGTCCATTCTC, ZO-1 reverse: AGCATCAGTTTCGGGTTTCC, ACTB forward: ATCGGCAATGAGCGGTTC, ACTB reverse: ACTGTGTTGGCATAGAGGTC, GAPDH forward: TTCAACGGCACAGTCAAGG, GAPDH reverse: CTCAGCACCAGCATCACC.

### Immunofluorescence microscopy

RBMEC were grown on collagen coated Labtek chambered slides (Nunc). Cells were fixed with 4% para*-*formaldehyde (PAF) on ice for 10 min, and permeabilised for 5 min with 0.1% Triton X-100 in PBS. The cells were then blocked in 10% normal goat serum diluted in PBS for 1 h. The primary antibodies were applied for 1 h at RT (PECAM-1: mouse, 1:50, Serotec; ZO-1: rabbit, 2.5 µg/mL, Zymed; Occludin: mouse IgG1-k 10 µg/mL, Zymed; Claudin-3: rabbit, 10 µg/mL, Zymed; Claudin-5: mouse, 10 µg/mL, Zymed). After 3 washes with PBS, the corresponding fluorescence-conjugated secondary antibody (rabbit-IgG: goat, 10 µg/mL, Invitrogen; mouse-IgG: goat, 10 µg/mL, Invitrogen) was added to the cells and incubated for 1 h. After 3 final washes, the slides were mounted with a glass coverslip with Dako Fluorescent Mounting Medium (DakoCytomation) and viewed with a fluorescence microscope.

### Electron microscopy

Transmission and scanning electron microscopy (TEM and SEM) was applied to characterize RBMEC cell layers. TEM and SEM images were generated according to the methods previously described [27].

### Statistical analysis

Statistical significances between the groups, which differed in the substance compositions, were calculated by an one-way ANOVA. For the comparison of groups with same substance compositions under different experimental transport conditions a two-way ANOVA was applied followed by an all pairwise multiple comparison procedure (Holm-Sidak method) with an overall significance level of 0.05. Data are presented as means ± SD (n = 3). ANOVAs and Spearman’s ranking order correlation coefficients were calculated by means of the software SigmaStat 3.5.

## Results

Three different models were used to study the transport of several NSAIDs (celecoxib, diclofenac, ibuprofen, meloxicam, piroxicam and tenoxicam) across the blood-brain barrier in vitro. The models based either on porcine cell line PBMEC/C1-2, on human cell line ECV304 or on a co-culture system consisting of primary rat brain microvascular endothelial cells (RBMEC) and astrocytes (AST). After single transport studies of each investigated NSAID across PBMEC/C1-2 and ECV304 cells, comprehensive group transport studies under different conditions were accomplished in order to study the influence of serum presence, glioma conditioned medium and addition of transporter inhibitors verapamil and probenecid. At last a group study across the RBMEC model was conducted in order to assess the transport ranking of the NSAIDs across of a model which is based on primary brain endothelial cells.

### Single transport studies across PBMEC/C1-2 and ECV304 layers

**Figure 1:**
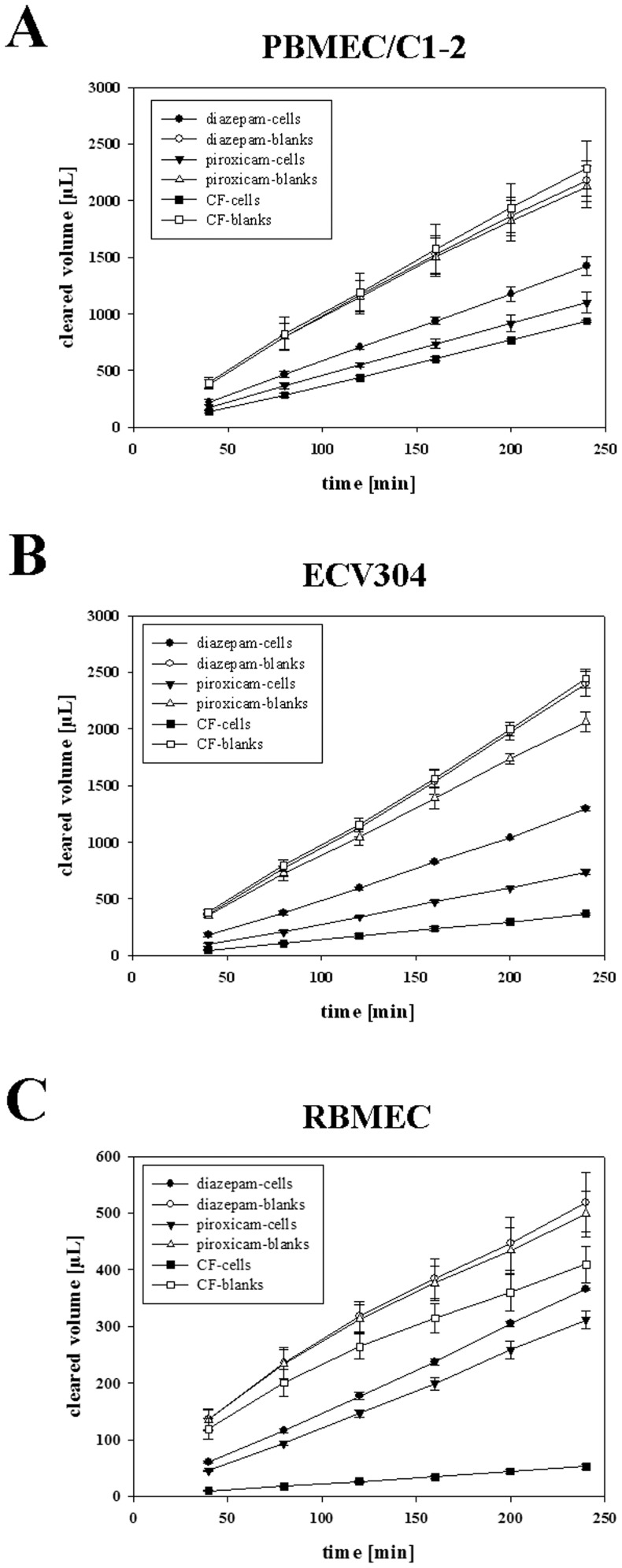
Time courses of single transport studies of piroxicam across PBMEC/C1-2, ECV304 and RBMEC layers. Comparison between the cleared volume vs. time graphs of internal standards for the transcellular transport route (diazepam, 100 µM), the paracellular transport route (carboxyfluorescein, 5 µM) and the NSAID piroxicam (100 µM) showed clearly that piroxicam permeated between the two markers across all three BBB (A: PBMEC/C1-2; B: ECV304; C: RBMEC) in vitro models. In addition, it was proved that as tighter the model is a wider frame between the two markers diazepam and carboxyfluorescein could be provided to analyze and compare permeabilities of single drugs. (n = 3 for each time point, data are presented as means ± SD).

First, transport studies with each NSAID (celecoxib, diclofenac, ibuprofen, meloxicam, piroxicam and tenoxicam) alone were accomplished across PBMEC/C1-2 or ECV304 cell layers. Diazepam was added as internal standard for the transcellular route, whereas carboxyfluorescein was supplemented as internal standard for the paracellular route. Figure 1 shows the time courses of the transport studies of piroxicam with diazepam and carboxyfluorescein across PBMEC/C1-2 (Figure 1A) and ECV304 (Figure 1B) cell layers. To compare these two cell line based models to the model based on RBMECs, the same transport experiment across RBMEC was additionally presented (Figure 1C). The linear transport of carboxyfluorescein confirmed that the cell layers of all three models remained stable during the entire transport experiment. Furthermore, the curves exemplified that the cells exhibited a significant barrier for the transport of investigated substances in comparison to the inserts without cells (blanks). However, different permeabilities for each substance across the inserts without cells pointed out the need of blank studies for each compound in order to be able to calculate the permeability coefficients across the cell layers only. As expected, slopes of the permeability time courses of piroxicam were between the slopes of the transcellular standard diazepam and the paracellular standard carboxyfluorescein. Total cleared volume values across RBMEC layers were lower in comparison to PBMEC/C1-2 and ECV304 because RBMEC studies were conducted with 12-well inserts, whereas PBMEC/C1-2 and ECV304 experiments were carried out with 6-well inserts. However, comparison of the frames of the cleared volume cell curves between diazepam and carboxyfluorescein revealed the smallest differences after 240 min for PBMEC/C1-2 (1.5-fold) followed by ECV304 (3.5-fold) and RBMEC (7-fold) reflecting the paracellular tightness of the applied layers. Results of all single studies across PBMEC/C1-2 and ECV304 layers were summarized in Table 1. Permeability coefficients (PE_cell_) of diazepam across PBMEC/C1-2 layers were in average between 32.29 and 58.93 µm/min, whereas of carboxyfluorescein between 16.67 and 19.66 µm/min. On the contrary, permeability coefficients of diazepam across ECV304 were between 19.93 and 39.95 µm/min, whereas of carboxyfluorescein between 3.89 and 5.67 µm/min. These data corresponded well with previous TEER and APTS-dextran data [23] confirming that ECV304 layers were significantly tighter than PBMEC/C1-2 layers and provide a broader window to distinguish between the permeabilities of single drugs. In order to account for cell layer’s variabilities permeability coefficients of NSAIDs were normalized with the corresponding permeability coefficient of diazepam. The resulting ratios to diazepam were used to rank the permeability of the single NSAIDs. Piroxicam was then the fastest substance across PBMEC/C1-2 layers followed by tenoxicam, ibuprofen, meloxicam, celecoxib and diclofenac. In case of the transport studies across ECV304 layers, piroxicam was also the fastest followed by ibuprofen, tenoxicam, celecoxib, meloxicam and diclofenac. Comparison of the rankings across PBMEC/C1-2 and ECV304 layers resulted in a high and significant Spearman’s ranking correlation coefficient of 0.929 (p<0.05).

**Table 1:** Single transport studies of NSAIDs across PBMEC/C1-2 and ECV304.

	substance	PS_blank_	PS_all_	PS_cell_	PE_all_	PE_cell_	Ratio to Diazepam*
		[µL/min]	[µL/min]	[µL/min]	[µm/min]	[µm/min]	
**PBMEC/C1-2**							
Piroxicam study	Piroxicam	8.69±0.72	4.63±0.36	9.91±1.60	11.02±0.87	23.82±3.81	0.55±0.05
	Diazepam	8.94±0.72	5.99±0.35	18.15±3.21	14.27±0.84	43.82±7.64	
	CF	9.42±0.94	4.01±0.09	7.00±0.28	9.56±0.22	16.67±0.67	
Ibuprofen study	Ibuprofen	6.72±0.12	3.45±0.15	7.10±0.65	8.22 ±0.36	16.95±1.54	0.46±0.01
	Diazepam	9.25±0.49	5.76±0.18	15.27±1.27	13.71±0.43	36.47±3.03	
	CF	11.69±0.30	4.47±0.08	7.24±0.21	10.65±0.19	17.25±0.49	
Meloxicam study	Meloxicam	8.03±1.21	3.15±0.10	5.18±0.28	7.50±0.25	12.34±0.68	0.41±0.13
	Diazepam	7.28±0.77	4.66±0.56	12.96±4.28	11.10±1.33	32.29±10.20	
	CF	10.09±1.18	4.17±0.07	7.12±0.19	9.94±0.16	16.95±0.46	
Tenoxicam study	Tenoxicam	7.47± 0.31	4.13±0.07	9.25±0.33	9.84±0.16	22.04±0.78	0.49±0.01
	Diazepam	9.17±0.47	6.17±0.10	18.86±0.94	14.69±0.24	44.95±2.24	
	CF	10.16±0.47	4.33±0.18	7.54±0.54	10.31±0.42	17.99±1.28	
Diclofenac study	Diclofenac	5.83±0.26	2.52±0.16	4.44±0.48	6.00±0.38	10.60±1.15	0.18±0.02
	Diazepam	8.00±0.12	6.04±0.10	24.69±1.74	14.39±0.25	58.93±4.14	
	CF	10.07±0.41	4.39±0.08	7.79±0.24	10.46±0.18	18.56±0.58	
Celecoxib study	Celecoxib	6.07±0.45	2.92±0.19	5.62±0.71	6.95±0.45	13.44±1.69	0.32±0.03
	Diazepam	8.64±0.44	5.79±0.36	17.50±3.20	13.78±0.86	42.30±7.61	
	CF	12.09±0.36	4.91±0.12	8.25±0.35	11.68±0.30	19.66±0.83	
**ECV304**							
Piroxicam study	Piroxicam	8.53±0.36	3.21±0.07	5.15±0.19	7.65±0.18	12.27±0.46	0.42±0.01
	Diazepam	10.13±0.43	5.57±0.02	12.39±0.11	13.26±0.05	29.49±0.27	
	CF	10.24±0.30	1.60±0.06	1.90±0.09	3.81±0.15	4.52±0.20	
Ibuprofen study	Ibuprofen	5.54±0.71	2.02±0.02	3.19±0.05	4.82±0.05	7.60±0.13	0.31±0.02
	Diazepam	8.21±0.29	4.60±0.13	11.43±0.68	10.94±0.32	24.86±1.63	
	CF	10.30±1.45	1.54±0.11	1.81±0.15	3.67±0.26	4.32±0.36	
Meloxicam study	Meloxicam	5.80±0.26	1.77±0.13	2.56±0.27	4.23±0.32	6.10±0.65	0.19±0.02
	Diazepam	7.49±0.45	4.81±0.14	13.40±1.14	11.44±0.34	32.00±2.71	
	CF	9.70±0.66	1.61±0.004	1.92±0.01	3.82±0.01	4.58±0.01	
Tenoxicam study	Tenoxicam	7.12±0.50	2.16±0.02	3.11±0.04	5.15±0.05	7.40±0.10	0.28±0.01
	Diazepam	8.92±0.72	4.97±0.08	11.21±0.41	11.83±0.19	26.71±0.98	
	CF	11.58±0.67	1.58±0.08	1.83±0.11	3.77±0.19	4.36±0.26	
Diclofenac study	Diclofenac	4.08±0.41	1.50±0.08	2.38±0.19	3.58±0.18	5.67±0.46	0.14±0.03
	Diazepam	6.46±1.01	4.66±0.15	16.67±1.88	11.09±0.36	39.95±4.48	
	CF	8.14±0.30	1.36±0.19	1.63±0.28	3.23±0.46	3.89±0.67	
Celecoxib study	Celecoxib	5.10±0.06	1.39±0.17	1.91±0.32	3.31±0.41	4.57±0.76	0.23±0.02
	Diazepam	10.20±1.48	4.58±0.34	8.32±1.12	10.91±0.80	19.93±2.67	
	CF	11.45±0.89	1.88±0.08	2.25±0.11	4.47±0.18	5.35±0.26	

* Ratio to Diazepam is calculated by average PE_cell_ data of the investigated NSAID and the corresponding diazepam value, CF  =  carboxyfluorescein.

Summary of permeability data of single transport studies with NSAIDs piroxicam, ibuprofen, meloxicam, tenoxicam, diclofenac and celecoxib across PBMEC/C1-2 as well as ECV304 cell layers. In each transport study one NSAID was applied together with the two permeability markers diazepam and carboxyfluorescein at the same time (n = 3, data are presented as means ± SD).

### Group transport studies across PBMEC/C1-2 and ECV304 layers

**Comparison to single studies.** No adverse effects onto the integrity of the barrier properties of PBMEC/1-2 as well as ECV304 layers were observed, although the number (and according applied concentration) of NSAIDs was increased from 1 (100 µM) to maximum 6 (600 µM) NSAIDs during the group studies. This was indicated by stable permeability values and linear curve progressions of the internal standard for the paracellular route carboxyfluoresein (tables 1 and 2). For example, permeability coefficients PE_cell_ for carboxyfluorescein were 16.67–19.66 µm/min in PBMEC/C1-2 single studies in comparison to 18.67±1.56 in the group study with all NSAIDs, and 3.89–5.35 µm/min in ECV304 single studies in comparison to 5.51±0.49 µm/min in the group study with all NSAIDs. Normalizing the permeability coefficients of the group study to diazepam yielded to following ranking across PBMEC/C1-2 layers: piroxicam, diazepam, ibuprofen, tenoxicam, meloxicam, diclofenac and celecoxib, where pairs piroxicam and diazepam, and tenoxicam and meloxicam penetrated almost equally fast. In case of ECV304 layers, diazepam was the fastest followed by piroxicam, ibuprofen, meloxicam, tenoxicam, diclofenac and celecoxib. Comparison of the rankings of the single and the group studies revealed a high Spearman’s rank order correlation coefficient of 0.893 (p<0.05) for PBMEC/C1-2 layers and of 0.821 (p<0.05) for ECV304 layers indicating that the group studies results showed similar permeability rankings. Minor deviations in the rankings may occur due to drug-drug interactions. Interestingly, comparison of the rankings of the group studies across PBMEC/C1-2 and ECV304 layers also revealed a high Spearman’s rank order correlation coefficient of 0.929 (p<0.05). These data indicated that high correlations in the rankings existed between the single and the corresponding group study, but also between the two different cell lines. Based on these results, it was decided to accomplish further group studies in order to investigate the influence of serum, glioma derived factors, the presence of carboxyfluorescein and meloxicam or of active transporter inhibitors probenecid and verapamil on the permeability properties of the studied NSAIDs.

**Table 2:** Group transport studies of NSAIDs across PBMEC/C1-2 and ECV304.

	PE_cell_	PE_cell_	PE_cell_	PE_cell_	PE_cell_	PE_cell_	PE_cell_	PE_cell_
	[µm/min]	[µm/min]	[µm/min]	[µm/min]	[µm/min]	[µm/min]	[µm/min]	[µm/min]
			serum-free medium	glioma conditioned			with Probenecid	with Verapamil
		without Celecoxib		medium	without CF	without Meloxicam	without Meloxicam	without Meloxicam
**substance:**								
Diazepam	40.04±2.99	24.91±2.57	36.65±2.71	39.18±2.32	33.96±3.53	52.96±5.83	39.12±4.26	58.03±5.10
Piroxicam	41.98±3.89	25.23±5.10	24.96±2.08	29.86±5.84	30.27±2.61	38.50±3.96	30.26±5.85	40.07±8.39
Ibuprofen	25.85±2.66	12.46±1.95	32.74±1.59	29.33±2.45	24.70±2.68	27.43±3.06	32.51±5.51	42.14±2.11
Meloxicam	22.87±2.64	9.39±0.33	23.71±1.20	24.32±1.18	19.97±2.72	n.a.*	n.a.*	n.a.*
Tenoxicam	23.10±1.18	16.69± 1.74	21.67±0.92	25.87±9.41	20.74±1.52	26.81±1.88	25.32±1.36	29.18±0.65
Diclofenac	19.50±1.59	10.25±0.80	30.20±2.45	19.82±1.45	17.22±1.63	20.46±1.91	25.80±2.77	32.33±1.65
Celecoxib	13.26±1.07	n.a.*	n.a.*	n.a.*	n.a.*	15.47±1.02	16.65±2.20	21.06±0.91
CF*	18.67±1.56	16.02±1.80	17.67±0.70	18.01±1.38	n.a.*	20.60±1.52	17.41±0.41	24.60±1.00
TEER [Ohm*cm^2^]	57.40±2.42	60.20±4.20	56.00±2.42	57.40±2.42	54.60±4.20	56.00±2.42	67.20±2.42	58.80±4.85
**substance:**								
Diazepam	22.74±3.12	23.93±2.16	36.72±4.71	38.44±3.27	26.46±2.33	40.69± 5.13	31.46±0.19	25.15± 2.47
Piroxicam	12.81± 2.69	17.15±1.00	22.70±2.03	18.50±0.93	18.20±1.51	27.52± 1.74	17.68±1.39	15.93± 0.93
Ibuprofen	10.81±1.07	14.24±0.57	28.04±2.44	8.59±0.45	12.05±1.42	16.85± 1.20	11.95±2.83	9.80± 2.37
Meloxicam	10.05±0.63	10.73±0.39	17.96±0.77	8.22±1.02	12.15±0.63	n.a.*	n.a.*	n.a.*
Tenoxicam	9.97± 0.46	9.86±0.09	11.55±2.94	8.23± 0.99	12.97±2.26	13.96± 0.80	3.79±0.84	11.91± 0.15
Diclofenac	7.70±0.58	9.02±0.50	24.29±1.77	5.93±0.91	9.65±0.97	12.62±0.89	10.53±0.92	7.24± 0.17
Celecoxib	6.58± 0.57	n.a.*	n.a.*	n.a.*	n.a.*	13.96± 0.41	9.31±0.60	6.20±0.19
CF*	5.51± 0.49	4.84±0.25	9.38±0.42	5.64±0.63	n.a.*	5.59±0.18	5.12±0.14	4.92± 0.15
TEER [Ohm*cm^2^]	121.8±2.42	134.4±2.42	123.3±2.42	124.6±2.42	127.4±4.85	128.8± 6.42	133.0±2.42	127.4± 6.42

* CF  =  carboxyfluorescein, n.a.  =  not added due to experimental or analytical reasons.

Summary of permeability data of group transport studies with NSAIDs piroxicam, ibuprofen, meloxicam, tenoxicam, diclofenac and celecoxib across PBMEC/C1-2 as well as ECV304 cell layers. In each transport study the two permeability markers diazepam and carboxyfluorescein were applied at the same time. The permeability of different group compositions (all, without celecoxib, without celecoxib and carboxyfluoresein, without meloxciam), in different transport media (all, serum-free medium, glioma-conditioned medium) and the added transporter inhibitors (without meloxciam with probenecid, without meloxicam with verapamil) was measured (n = 3, data are presented as means ± SD).

**Figure 2:**
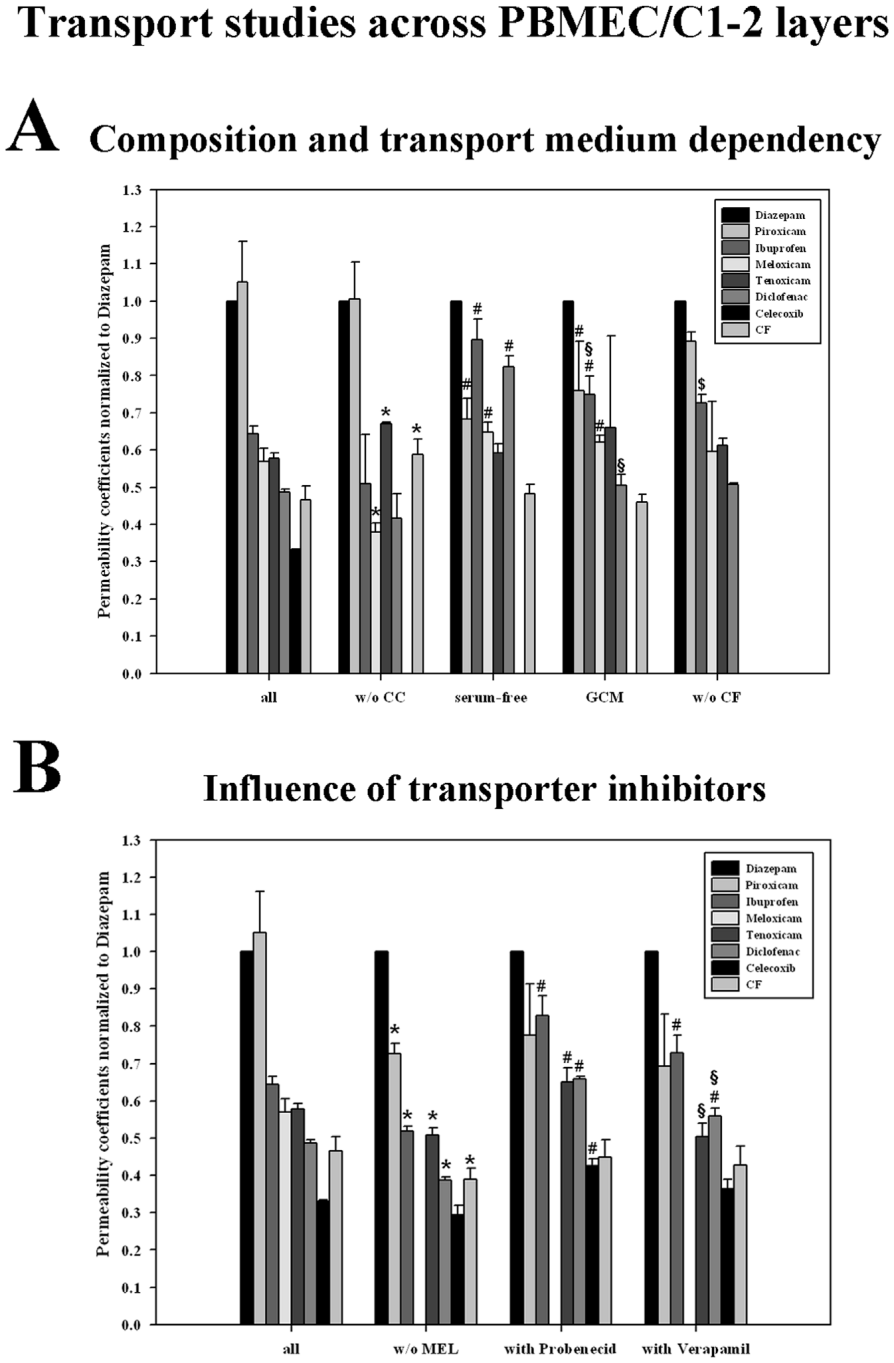
Rankings of the group transport studies with NSAIDs across PBMEC/C1-2 layers. Permeability coefficient of each substance was normalized to the corresponding permeability coefficient of internal standard diazepam of the same experiment. **A):** Variant substance compositions - results of the group study with all investigated substances (diazepam, piroxicam, ibuprofen, meloxicam, tenoxicam, diclofenac, celecoxib, carboxyfluorescein = CF) were compared to the study without celecoxib (w/o CC), without celecoxib accomplished in serum-free C6 medium (serum-free), without celecoxib accomplished in PBMEC-Fib medium (GCM  =  glioma conditioned medium) and without celecoxib and carboxyfluorescein (w/o CF). **B):** Different transport study conditions - results of the group study with all investigated substances (diazepam, piroxicam, ibuprofen, meloxicam, tenoxicam, diclofenac, celecoxib, carboxyfluorescein = CF) were compared to the study without meloxicam (w/o MEL), without meloxicam and with probenecid (with Probenecid) and without meloxicam and with verapamil (with Verapamil). To calculate the statistical significances between the groups, which differed in the substance compositions, a one-way ANOVA was used, to compare the groups with same substance compositions under different experimental transport conditions (in A: w/o CC, serum-free medium, GCM; in B: w/o MEL, with Probenecid, with Verapamil) a two-way ANOVA was accomplished followed by an all pairwise multiple comparison procedure (Holm-Sidak method) with an overall significance level of 0.05. Statistical significance (p<0.05) for each substance is indicated in the figure by * (all vs. w/o CC, all vs. w/o MEL), by # (w/o CC vs. serum-free or GCM; w/o MEL vs. with Probenecid or with Verapamil), by § (serum-free vs. GCM; with Probenecid vs. with Verapamil) or by $ (w/o CC vs. w/o CF). Data are presented as means ± SD (n = 3).

**Influence of serum.** In order to test the influence of the presence of serum, group studies were conducted with NSAIDs piroxicam, ibuprofen, meloxicam, tenoxicam and diclofenac in serum containing and in serum-free transport medium across PBMEC/C1-2 and ECV304 layers. Celecoxib was excluded in this set-up due to analytical reasons. Because of the exclusion of celecoxib, a group study in serum-containing medium without celecoxib was accomplished as a control study for the corresponding serum-free group study. Comparison of group studies accomplished in serum-containing medium with and without celecoxib revealed significant differences and underlined possible drug-drug interactions of celecoxib with the transport of the other applied compounds (table 2). In case of PBMEC/C1-2 layers, analyzing the normalized data permeability for meloxicam decreased from 0.57±0.04-fold to 0.38±0.03-fold, whereas permeability for tenoxicam (0.58±0.01 to 0.67±0.00) and carboxyfluorescein (0.46±0.04 to 0.59±0.04) increased significantly (fig.2). On the contrary, exclusion of celecoxib during the study across ECV304 layers increased only the permeability of ibuprofen (0.48±0.05 to 0.60±0.03) significantly. These different effects by celecoxib exclusion were reflected in a non-significant Spearman’s rank order correlation coefficient of 0.5 (p = 0.217) comparing the changed ranking of the group studies across PBMEC/C1-2 and ECV304 without celecoxib.

**Figure 3:**
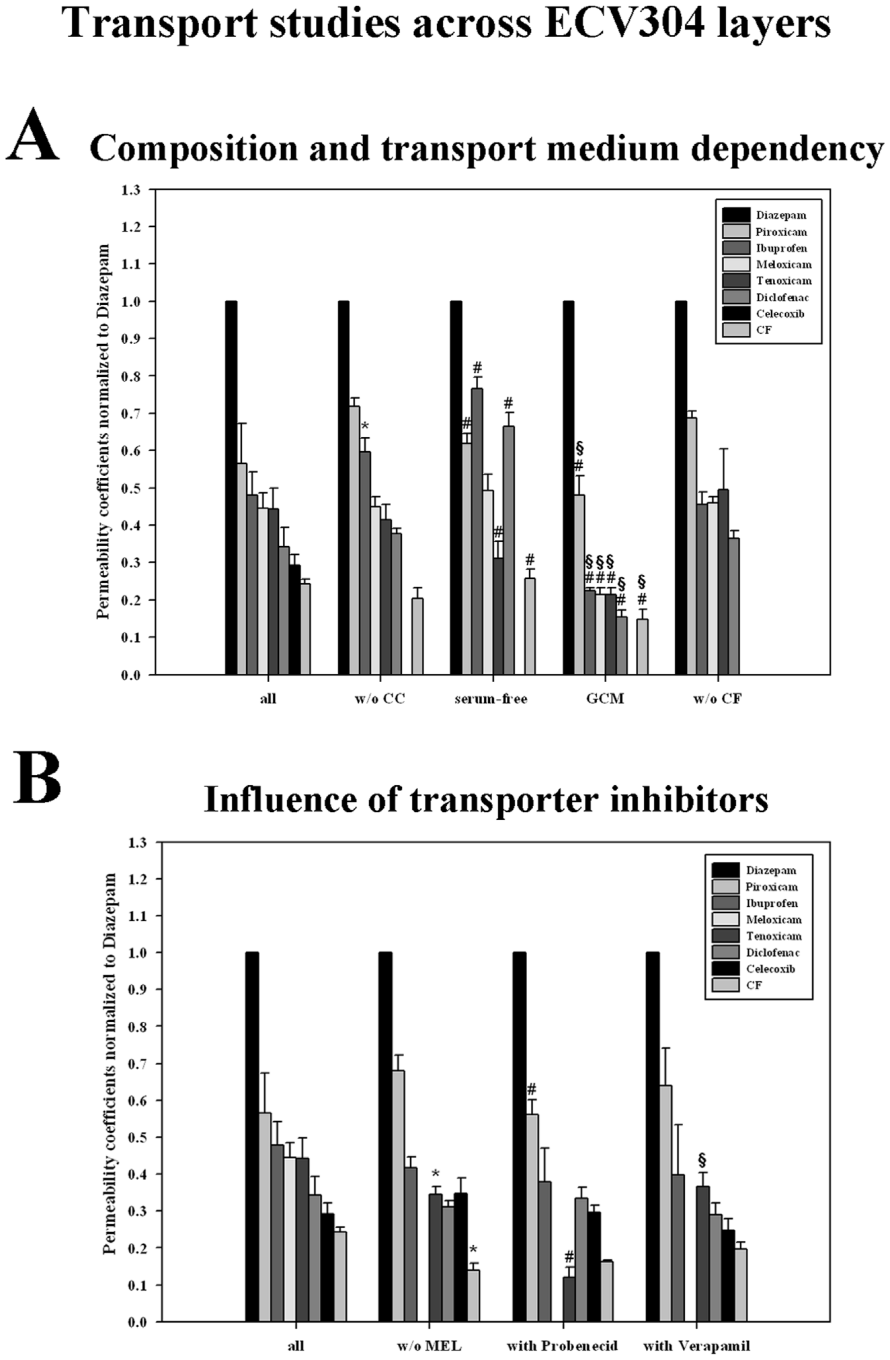
Rankings of the group transport studies with NSAIDs across ECV304 layers. Permeability coefficient of each substance was normalized to the corresponding permeability coefficient of internal standard diazepam of the same experiment. **A):** Variant substance compositions - results of the group study with all investigated substances (diazepam, piroxicam, ibuprofen, meloxicam, tenoxicam, diclofenac, celecoxib, carboxyfluorescein = CF) were compared to the study without celecoxib (w/o CC), without celecoxib accomplished in serum-free C6 medium (serum-free), without celecoxib accomplished in PBMEC-Fib medium (GCM  =  glioma conditioned medium) and without celecoxib and carboxyfluorescein (w/o CF). **B):** Different transport study conditions - results of the group study with all investigated substances (diazepam, piroxicam, ibuprofen, meloxicam, tenoxicam, diclofenac, celecoxib, carboxyfluorescein = CF) were compared to the study without meloxicam (w/o MEL), without meloxicam and with probenecid (with Probenecid) and without meloxicam and with verapamil (with Verapamil). To calculate the statistical significances between the groups, which differed in the substance compositions, a one-way ANOVA was used, to compare the groups with same substance compositions under different experimental transport conditions (in A: w/o CC, serum-free medium, GCM; in B: w/o MEL, with Probenecid, with Verapamil) a two-way ANOVA was accomplished followed by an all pairwise multiple comparison procedure (Holm-Sidak method) with an overall significance level of 0.05. Statistical significance (p<0.05) for each substance is indicated in the figure by * (all vs. w/o CC, all vs. w/o MEL), by # (w/o CC vs. serum-free or GCM; w/o MEL vs. with Probenecid or with Verapamil) or by § (serum-free vs. GCM; with Probenecid vs. with Verapamil). Data are presented as means ± SD (n = 3).

Absence of serum resulted in increased absolute permeability coefficients for almost all investigated substances in both BBB models (table 2, except piroxicam across PBMEC/C1-2). In comparison to the group study without celecoxib, normalized permeability of piroxicam (1.01±0.09 to 0.68±0.06) decreased significantly, whereas ibuprofen (0.51±0.13 to 0.89±0.06), diclofenac (0.42±0.07 to 0.82±0.03) and meloxicam (0.38±0.03 to 0.65±0.03) migrated faster across the PBMEC/C1-2 layers (fig.2). In case of cell line ECV304, piroxicam (0.72±0.02 to 0.62±0.03) and tenoxicam (0.41±0.04 to 0.31±0.04) permeated significantly slower, but ibuprofen (0.60±0.04 to 0.77±0.03), diclofenac (0.38±0.01 to 0.66±0.04) and carboxyfluorescein (0.20±0.03 to 0.26±0.02) got also significantly faster when excluding serum in the experimental set-up (fig.3). Comparing the rankings only one significant correlation was obtained between results of the study without celecoxib and the test in the serum-free medium. Interestingly, the absolutely same permeability ranking was found for the studies without serum across PBMEC/C1-2 and ECV304 layers (Spearman’s ranking order correlation coefficient  =  1). In both cases, diazepam was the fastest substance followed by ibuprofen, diclofenac, piroxicam, meloxicam, tenoxicam and carboxyfluorescein.

In order to elucidate the role of serum binding of the tested substances during these transport studies, serum binding was measured by an ultrafiltration method. Results in the serum-containing transport medium (7.5% serum) showed that meloxicam (20.43%), diclofenac (52.61%) and ibuprofen (42.75%) exhibited the highest serum binding values which was concordant to their increased permeability ranking in the studies conducted with serum-free medium (table 3).

**Table 3:** Serum binding [%] of NSAIDs in transport media.

substance	Serum concentration		
	7.5%	50%	100%
Diazepam	8.17±0.49	19.52±0.13	22.52±0.06
Piroxicam	11.30±1.05	61.19±0.79	81.92±0.06
Ibuprofen	42.75±1.41	90.52±0.09	92.82±0.003
Meloxicam	20.43±1.35	75.14±0.23	84.16±0.03
Tenoxicam	11.19±1.81	56.32±1.13	78.47±0.03
Diclofenac	52.61±1.36	89.38±0.05	90.57±0.02
CF*	17.72±4.57	34.39±8.87	45.84±1.15

* CF  =  carboxyfluorescein.

Serum binding [%] of NSAIDs, diazepam and carboxyfluorescein. Serum binding was assessed using same substance group compositions as applied for group transport studies with C6 medium containing either 0% or 7.5% serum. Data were presented for serum amounts of 7.5, 50 and 100% (n = 3, means ± SD).

**Influence of glioma conditioned medium.** It was reported in several previous studies that glioma derived factors can alter the properties of BBB in vitro models significantly [28]–[30]. This was also shown for cell lines PBMEC/C1-2 and ECV304 [23], [31]. All transport studies until now were accomplished in basal C6 medium. Consequently, we were interested whether the usage of glioma conditioned medium (of this basal C6 medium) may influence the permeability of the NSAIDs investigated. Comparison of the transport studies without celecoxib and the studies in glioma conditioned medium resulted in significant differences of the permeability behaviour of the tested drugs. Considering normalized data, piroxicam (1.01±0.09 to 0.76±0.13) permeated slower and ibuprofen (0.51±0.13 to 0.75±0.05) as well as meloxicam (0.38±0.03 to 0.62±0.02) migrated faster across PBMEC/C1-2 layers (fig.2). In case of ECV304 cells, after normalization to diazepam all NSAIDs (piroxicam, ibuprofen, meloxicam, tenoxicam and diclofenac) but also the paracellular marker carboxyfluorescein penetrated significantly slower in comparison to the study without celecoxib (fig.3). Interestingly, transport studies of NSAIDs in glioma conditioned medium revealed the same ranking across PBMEC/C1-2 and ECV304 layers: diazepam was the fastest followed by piroxicam, ibuprofen, tenoxicam, meloxicam, diclofenac and caroboxyfluorescein. The corresponding Spearman’s ranking order correlation coefficient was 0.964 (p<0.05). Comparing the rankings of the glioma conditioned medium study across PBMEC/C1-2 layers with the corresponding control study in the basal C6 medium resulted in no significant correlation (p>0.05), whereas in case of ECV304 layers this comparison showed a significant Spearman’s ranking order correlation coefficient of 0.964 (p<0.05).

**Influence of carboxyfluorescein.** The exclusion of carboxyfluoresein was done in order to investigate its possible influence onto the permeability properties of the NSAIDs. Comparison of these group studies with the group studies without celecoxib revealed only one significant change. The exclusion of carboxyfluorescein increased the normalized permeability of ibuprofen (0.51±0.13 to 0.73±0.02) in the PBMEC/C1-2 study significantly. However, no significant ranking order correlation was found for the comparisons of the studies without celecoxib and carboxyfluorescein.

**Influence of inhibitors probenecid and verapamil.** In order to test the influence of classical transporter inhibitors probenecid, meloxicam had to be excluded from the NSAID mix due to analytical reasons. Consequently, the differences between the group study with all NSAIDs vs. all NSAIDs excluded meloxicam were studied. Interestingly, the absence of meloxicam resulted in a distinct increase of the absolute permeability coefficients in both models (table 2). On the contrary, analyzing the permeability coefficients normalized to diazepam, the permeability of all NSAIDs except celecoxib was significantly decreased across PBMEC/1-2 layers (fig. 2B), whereas only tenoxicam and carboxyfluorescein decreased across ECV304 layers (fig. 3B).

Comparison of normalized data the group studies without meloxicam vs. with probenecid revealed significant increases of ibuprofen (0.52±0.01 to 0.83±0.05), tenoxicam (0.51±0.02 to 0.65±0.04), diclofenac (0.39±0.00 to 0.66±0.00) and celecoxib (0.29±0.03 to 0.43±0.02) across PBMEC/C1-2, but significant decreases of piroxicam (0.68±0.04 to 0.56±0.04) and tenoxicam (0.35±0.02 to 0.12±0.03) across ECV304 layers. However, for all four group studies a significant Spearman’s ranking order correlation coefficient was found (PBMEC/C1-2 without meloxicam vs. without melocicam, but with probenecid: 0.857, p<0.05; ECV304 without meloxicam vs. without melocicam, but with probenecid: 0.75, p<0.05; PBMEC/C1-2 without meloxicam vs. ECV304 without meloxicam: 0.857, p<0.05; PBMEC/C1-2 without melocicam, but with probenecid vs. ECV304 without melocicam, but with probenecid: 0.821, p<0.05).

The comparison of normalized data of group studies without meloxicam vs. with verapamil showed significant increases of the permeability of ibuprofen (0.52±0.01 to 0.73±0.05) and diclofenac (0.39±0.00 to 0.56±0.02) across PBMEC/C1-2, but no significant change across ECV304 layers. However, in this case a significant Spearman’s ranking order correlation coefficient was obtained for all group studies (PBMEC/C1-2 without meloxicam vs. without melocicam, but with verapamil: 0.857, p<0.05; ECV304 without meloxicam vs. without melocicam, but with verapamil: 0.929, p<0.05; PBMEC/C1-2 without melocicam, but with verapamil vs. ECV304 without melocicam, but with verapamil: 0.786, p<0.05).

### Group transport study across primary RBMEC layers

**Figure 4:**
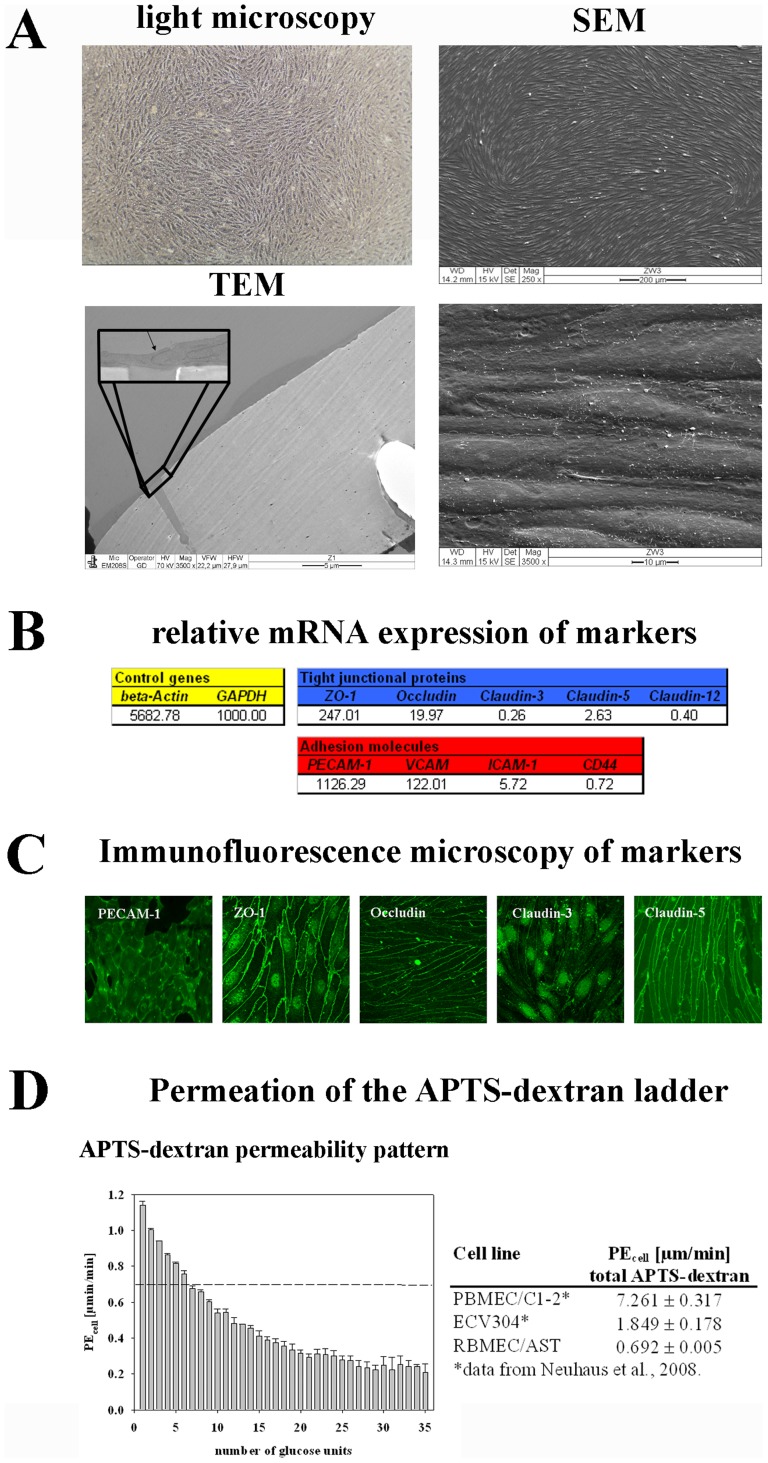
Characterization of the BBB model based on primary rat brain microvascular endothelial cells (RBMEC) and astrocytes. RBMECs grow in endothelial cell typical spindle-like morphology proven by light and scanning electron microscopy (SEM). Transmission electron microscopic (TEM) images confirmed that RBMEC grow as a monolayer. The enlarged part of the image shows two RBMECs connected to each other directly over a pore of the Transwell insert membrane (A). mRNA expressions of tight junction proteins ZO-1, occludin, claudin-3, claudin-5 and claudin-12, and of adhesion molecules PECAM-1, VCAM, ICAM-1 and CD44. All data were related to endogenous control GAPDH which was set to 1000 (B). Immunofluorescence images of PECAM-1, ZO-1, occludin, claudin-3 and claudin-5 confirmed the protein’s presence and localization in RBMEC layers (C). Transport studies with paracellular marker APTS-dextran ladder confirmed functionality of the barrier. RBMEC layers were able to differentiate between the different dextran fractions in a molecular size-dependent manner. Comparison of the permeability coefficients for APTS-dextran across PBMEC/C1-2, ECV304 and RBMEC layers is presented in the table on the right side (D).

Tightness characterization of cell layers used in BBB in vitro models is essential for the interpretation of transport study results. The tightness of a cell layer can be described by TEER or the permeability of a paracellular marker. The tightness properties of the BBB in vitro model consisting of the RBMEC/AST co-culture have not been characterized until now. Consequently, the RBMEC cell layers and their tightness properties had to be investigated prior drug transport studies. This was necessary in order to be able to estimate the role of the passive, paracellular transport route for NSAIDs across RBMEC cell layers in comparison to PBMEC/C1-2 and ECV304 layers. The results were summarized in figure 4. Light microscopic as well as scanning electron microscopic (SEM) images confirmed the spindle-like morphology of the RBMEC layers. Transmission electron microscopy (TEM) proved that RBMECs grew as monolayers under the established conditions (fig. 4A). At the mRNA level, presence and the amount of major BBB tight junction proteins occludin, ZO-1, claudin-3, claudin-5 and claudin-12 was quantified by RT-qPCR. In addition to tight junctional proteins, expression of endothelial markers (adhesion molecules) PECAM-1, VCAM, ICAM-1 and CD44 was assessed and confirmed (fig. 4B). Furthermore, the presence and correct localisation of PECAM-1, ZO-1, occludin, claudin-3 and claudin-5 were also confirmed by immunofluorescence microscopy at the protein level (fig. 4C). In order to describe functional tightness, transport studies with the paracellular marker APTS-dextran were accomplished. Figure 4D shows on the left side a typical pattern of the APTS-dextran ladder after a transport study, which confirmed that the fractions with increasing glucose units permeated slower than those with fewer glucose units indicating a molecular size dependent paracellular permeability. On the right side a summary of APTS-dextran permeability coefficients is given. Comparison of the data pointed out that RBMEC layers were approximately 2.5-fold tighter than ECV304 and 10-fold tighter than PBMEC/C1-2 layers underlining the suitability of RBMEC layers for drug transport studies concerning the tightness of the layers.

After validation of RBMEC layers a transport group study with NSAIDs piroxicam, ibuprofen, meloxicam, tenoxicam and diclofenac was accomplished and resulted in following permeability ranking: diazepam was the fastest followed by ibuprofen and piroxicam (which were equally fast), diclofenac, meloxicam and tenoxicam (table 4). This ranking was compared with the rankings of the transport studies across PBMEC/C1-2 and ECV304 layers in glioma conditioned medium, because the RBMEC study was conducted in astrocyte conditioned medium. However, no significant Spearman’s ranking order correlation was found between the RBMEC and either the PBMEC/C1-2 or the ECV304 model (correlation coefficient = 0.715, p = 0.136 in both cases).

**Table 4:** Group transport study of NSAIDs across RBMEC.

	substance	PS_blank_	PS_all_	PS_cell_	PE_all_	PE_cell_	Ratio to Diazepam*
		[µL/min]	[µL/min]	[µL/min]	[µm/min]	[µm/min]	
**RBMEC**							
group study	Diazepam	1.93±0.13	1.29±0.02	3.77±0.16	14.35±0.20	41.92±1.75	1.00±0.00
	Piroxicam	1.94±0.12	1.15±0.03	2.82±0.17	12.76±0.33	31.39±1.94	0.76±0.04
	Ibuprofen	1.71±0.15	1.07±0.06	2.86±0.39	11.88±0.62	31.99±4.28	0.76±0.09
	Meloxicam	1.86±0.12	0.89±0.02	1.73±0.09	9.97±0.27	19.27±0.99	0.47±0.02
	Tenoxicam	1.74±0.10	0.70±0.02	1.18±0.05	7.82±0.21	13.12±0.59	0.32±0.01
	Diclofenac	1.65±0.13	0.96±0.05	2.39±0.27	10.84±0.51	26.73±2.97	0.64±0.06

* Ratio to Diazepam is calculated by average PE_cell_ data of the investigated NSAID and the corresponding diazepam value.

Summary of permeability data of the group study with NSAIDs piroxicam, ibuprofen, meloxicam, tenoxicam and diclofenac across the RBMEC cell layers (n = 3, data are presented as means ± SD).

## Discussion

The transport of NSAIDs across BBB in vitro models was investigated in the presented work. Two major aspects of this study should be emphasized. On the one hand no systematic study about the permeability of several NSAIDs across the blood-brain barrier (BBB) in vitro is available, although it is known that many NSAIDs are able to penetrate into the CNS in vivo [15]–[20]. Furthermore, NSAIDs could act beneficially in diseases such as Alzheimers disease, epilepsy or traumatic brain injury, for which alterations of BBB functionality were shown [5], [9], [11], [18], [32]. Consequently, found BBB permeability ranking orders of NSAIDs in this work may help to estimate the role of BBB permeability with regard to their CNS effects. On the other hand rankings were assessed using different study conditions. Especially changes of ranking orders of studies with different experimental media underlined the importance of the used experimental medium. In order to give a comprehensive overview three different in vitro BBB models derived from three different species (porcine, human, rat) with distinct different tightness properties were applied to study the transport of several NSAIDs (celecoxib, diclofenac, ibuprofen, meloxicam, piroxicam and tenoxicam).

Single substance studies with both cell lines revealed a transport ranking which showed a high correlation to corresponding group studies. As previously reported, normalization to internal standard diazepam can be used to account for cell layer’s variabilities, which is especially valueable for studies with cell layers of moderate tightness [23], [27], [33]. Minor deviations in the rankings between single and group studies might be explained by drug-drug interactions. Interestingly, diclofenac outran celecoxib in the group studies in both cell line models. Celecoxib was reported to have the potential to block abcg2 (bcrp) functionality and thus might increase permeability of abcg2 substrate diclofenac. Furthermore, celecoxib could inhibit abcc4 (mrp4), to which meloxicam can bind [34]–[38]. This may support that meloxicam got ahead of celecoxib in the ECV304 group study in comparison to corresponding single study. In summary, these facts could explain the minor changes in the rankings between single and group studies. Rare data of NSAID permeability across other cell models are available. In comparison to one study with Caco-2 cells, ranking across the BBB models (piroxicam –ibuprofen – meloxicam – diclofenac) was distinctly different to the ranking across Caco-2 cell layers (piroxicam – diclofenac – meloxicam – ibuprofen) [39]. Although drug-drug interactions probably occur, group studies – also termed as cocktail studies – have proved to be of high value for drug screenings [33], [40]–[42]. Considering deviations of permeability coefficients of transcellular marker diazepam one major advantage of group studies is to obtain permeability rankings of drugs which migrated across the same cell layers. Therefore, it was decided to conduct further studies as group studies to investigate the influence of varying experimental medium, group compositions and addition of transporter inhibitors (verapamil, probenecid).

In general, significant congruence was obtained between group study rankings obtained from the BBB in vitro models based on PBMEC/C1-2 and on ECV304 cells. Lack of serum in experimental medium revealed same rankings across both models. Notably, with ibuprofen and diclofenac both substances containing carboxylic acid groups migrated significantly faster in both models. In this context, Parepally et al. (2006) reported a distinctly decreased BBB permeability of ibuprofen with increasing amount of plasma proteins [15]. Furthermore, they showed a saturated transport profile for ibuprofen in studies without plasma proteins suggesting involvement of an active transport system for ibuprofen across the BBB. Our studies without serum confirmed these data pointing to the important role of serum during drug transport studies due to different drug-serum binding and consequent lowered free drug concentrations. This was supported by our measured serum binding values especially for ibuprofen, diclofenac and meloxicam. In this case, significantly reduced amounts of unbound drugs in the transport medium containing 7.5% serum probably led to decreased permeability rates and minor positions in the permeability rankings within the investigated groups. However, it should not be forgotten that serum itself could also influence directly cell layer’s properties and consequently also drug transport processes.

Studies with GCM highlighted the influence of astrocyte derived factors on transport studies across BBB models. Interestingly, changes in transport rankings in comparison to group studies in C6 medium without celecoxib led to the same transport rankings in both models. This finding seems to be very important considering the experimental set-up of transport studies and the role of astrocytes for barrier properties of BBB in vitro models. Many research groups are using simplified transport buffer systems mainly to ease drug analysis or achieve improved data comparability. Our data imply distinct changes of barrier properties for drug transport depending on the experimental transport buffer. Even preincubation of 30–60 minutes and subsequent four hours lasting experiments were enough to result in significant differences of permeability coefficients. Considering our data and the importance of astrocyte derived soluble factors for improved barrier tightness in BBB models [28]–[30], it could be recommended to accomplish studies in growth medium as long as the experimental and analytical set-up allows it.

Although carboxyfluorescein had been widely used as a paracellular marker molecule, it had also been reported being a substrate for mrp and organic anion transporter (oat) [23], [43]–[45]. Consequently, we have carried out group transport studies excluding carboxyfluorescein. Notably, only permeability of ibuprofen, which was reported to inhibit oat and mrp activity [38], [46], was increased across PBMEC/C1-2 layers. Hence, it could be supposed that interaction of carboxyfluorescein and ibuprofen at mrps or oats was responsible for this effect. In order to estimate the role of active transporters during these group transport studies general transporter inhibitors probencid and verapamil were added. Probenecid had been published as a well-known blocker of mrp1/2 (abcc1 and abcc2) and oats, whereas verapamil had been used as competitive inhibitor of P-gp (abcb1) [46]–[48] **.** Addition of probenecid increased normalized permeability of ibuprofen, tenoxicam, diclofenac and celecoxib in the PBMEC/C1-2 model, whereas decreased piroxicam and tenoxicam migration in the ECV304 model suggesting very complex interactions. It was reported that ibuprofen and diclofenac inhibited mrp2, mrp4, oat1 and oat3, celecoxib blocked mrp1 and mrp4 activity and piroxicam acted as mrp2 and mrp4 inhibitor [34], [37], [38], [46]. Thus, it could be assumed that interactions at mrps and oats might lead to these changes. Addition of verapamil increased only the normalized permeability of ibuprofen and diclofenac in the PBMEC/C1-2 model, although it was shown that celecoxib can decrease P-gp activity just as diclofenac and ibuprofen [34], [49], [50]. In summary, it was shown that permeability of every investigated NSAID was altered by addition either of transport blockers, changed transport medium or altered substance composition.

At last a NSAID group study across a BBB model based on co-culture of primary rat endothelial cells with astrocytes was carried out. In comparison to the PBMEC/C1-2 and the ECV304 model, the RBMEC/AST model exhibited significantly higher paracellular tightness reflected in low APTS-dextran permeability and high TEER values. In addition to the high tightness, RBMEC/AST were chosen in order to include a model derived from a rodent species, in particular from rat, since pig (PBMEC/C1-2) and human (ECV304) are genetically very cognate [51]. Characterisation of the model proved monolayer structure, major tightness properties and endothelial marker expression confirming suitability of this model as BBB model. In general, similar tendencies were shown as compared to transport results obtained with PBMEC/C1-2 and ECV304 models. For example, ibuprofen and piroxicam were the fastest after diazepam. However, moderate differences were found. In particular ibuprofen overran piroxicam, and diclofenac overran meloxicam and tenoxicam, which in the end led to no significant ranking correlation between the primary rat RBMEC/AST model and the porcine PBMEC/C1-2 and human ECV304 model. In this context, it should be considered that species specific properties might be responsible for these differences.

## Conclusion

NSAIDs permeated across used BBB in vitro models well confirming in vivo data about BBB permeability and CNS side effects of NSAIDs. High correlations were found between transport rankings of NSAIDs across both PBMEC/C1-2 and ECV304 cell layers. Group transport studies revealed similar results as single substance studies yielding in high correlation coefficients which confirmed usefulness of the application of group studies. Varying transport medium underlined the impact of the experimental transport buffer on the results and led to the recommendation to use astrocyte conditioned medium for transport studies. Altered compositions of studied NSAID groups and addition of transporter protein inhibitors showed that their transport were regulated by drug-drug interactions, presumably at the site of drug transporter proteins. In conclusion, the presented work provides a comprehensive overview of transport rankings of NSAIDs across three BBB in vitro models and highlighted the influence of the experimental conditions on these transport rankings.

## Acknowledgments

We are very thankful to Regina Lauer for the establishment of the PBMEC/C1-2 model and providing the basics of HPLC analysis.

## References

[1] Joo F (1996) Endothelial cells of the brain and other organ systems: some similarities and differences. Prog Neurobiol 48: 255–273. 8735879 10.1016/0301-0082(95)00046-1

[2] Neuhaus W, Noe CR (2009) Transport at the Blood–Brain Barrier. In: Ecker G, Chiba P, editors. Transporters as Drug Carriers: Structure, Function, Substrates: 44 (Methods and Principles in Medicinal Chemistry). Weinheim, Germany: Wiley- VCH Verlag GmbH & Co. KGaA; 2009. pp. 263–298.

[3] Abbott NJ, Rönnbäck L, Hansson E (2006) Astrocyte-endothelial interactions at the blood-brain barrier. Nat Rev Neurosci 7: 41–53. 16371949 10.1038/nrn1824

[4] Cucullo L, Hossain M, Tierney W, Janigro D (2013) A new dynamic in vitro modular capillaries-venules modular system: cerebrovascular physiology in a box. BMC Neurosci 6: 14–18. 10.1186/1471-2202-14-18 PMC3598202 23388041

[5] Bell R, Zlokovic B (2009) Neurovascular mechanisms and blood–brain barrier disorder in Alzheimer's disease. Acta Neuropathol 118: 103–113. 19319544 10.1007/s00401-009-0522-3 PMC2853006

[6] Kortekaas R, Leenders KL, van Oostrom JC, Vaalburg W, Bart J, et al (2005) Blood–brain barrier dysfunction in parkinsonian midbrain in vivo. Ann Neurol 57: 176–179. 15668963 10.1002/ana.20369

[7] Minagar A, Alexander JS (2003) Blood–brain barrier disruption in multiple sclerosis. Mult Scler 9: 540–549. 14664465 10.1191/1352458503ms965oa

[8] Kleinschnitz C, Blecharz K, Kahles T, Schwarz T, Kraft P, et al (2011) Glucocorticoid insensitivity at the hypoxic blood–brain barrier can be reversed by inhibition of the proteasome. Stroke 42: 1081–1089. 21330632 10.1161/STROKEAHA.110.592238

[9] Thal SC, Schaible EV, Neuhaus W, Scheffer D, Brandstetter M, et al (2013) Inhibition of proteasomal glucocorticoid receptor degradation restores dexamethasone-mediated stabilization of the blood-brain barrier after traumatic brain injury. Crit Care Med 41: 1305–1315. 23474678 10.1097/CCM.0b013e31827ca494

[10] Zlokovic BV (2011) Neurovascular pathways to neurodegeneration in Alzheimer's disease and other disorders. Nat Rev Neurosci 12: 723–738. 22048062 10.1038/nrn3114 PMC4036520

[11] Dokmeci D (2004) Ibuprofen and Alzheimer's disease. Folia Med (Plovdiv) 46: 5–10. 15506544

[12] Mutschler E, Geisslinger G, Kroemer HK, Menzel S, Ruth P (2012) Mutschler Arzneimittelwirkungen: Pharmakologie - Klinische Pharmakologie – Toxikologie. Stuttgart: Wissenschaftliche Verlagsgesellschaft Stuttgart.

[13] Smolinske SC, Hall AH, Vandenberg SA, Spoerke DG, McBride PV (1990) Toxic effects of nonsteroidal anti-inflammatory drugs in overdose. An overview of recent evidence on clinical effects and dose-response relationships. Drug Saf 5: 252–274. 2198051 10.2165/00002018-199005040-00003

[14] Aygün D, Kaplan S, Odaci E, Onger ME, Altunkaynak ME (2012) Toxicity of non-steroidal anti-inflammatory drugs: a review of melatonin and diclofenac sodium association. Histol Histopathol 27: 417–436. 22374720 10.14670/HH-27.417

[15] Parepally JM, Mandula H, Smith QR (2006) Brain uptake of nonsteroidal antiinflammatory drugs: ibuprofen, flurbiprofen, and indomethacin. Pharm Res 23: 873–881. 16715377 10.1007/s11095-006-9905-5

[16] Dembo G, Park SB, Kharasch ED (2005) Central nervous system concentrations of cyclooxygenase-2 inhibitors in humans. Anesthesiology 102: 409–415. 15681959 10.1097/00000542-200502000-00026

[17] Jolliet P, Simon N, Brée F, Urien S, Pagliara A, et al (1997) Blood-to-brain transfer of various oxicams: effects of plasma binding on their brain delivery. Pharm Res 14: 650–656. 9165538 10.1023/a:1012165414610

[18] Hakan T, Toklu HZ, Biber N, Ozevren H, Solakoglu S, et al (2010) Effect of COX-2 inhibitor meloxicam against traumatic brain injury-induced biochemical, histopathological changes and blood-brain barrier permeability. Neurol Res 32: 629–635. 19660237 10.1179/016164109X12464612122731

[19] Foster KA, Weiss M, Roberts MS (2002) Distribution kinetics of solutes in the isolated in-situ perfused rat head using the multiple indicator dilution technique and a physiological two-barrier model. J Pharm Pharmacol 54: 373–382. 11902803 10.1211/0022357021778619

[20] Farooq F, Abadía-Molina F, Mackenzie D, Hadwen J, Shamim F, et al (2013) Celecoxib increases SMN and survival in a severe spinal muscular atrophy mouse model via p38 pathway activation. Hum Mol Genet 22: 3415–3424. 23656793 10.1093/hmg/ddt191

[21] Teifel M, Friedl P (1996) Establishment of the permanent microvascular endothelial cell line PBMEC/C1-2 from porcine brains. Exp Cell Res 228: 50–57. 8892970 10.1006/excr.1996.0298

[22] Neuhaus W, Bogner E, Wirth M, Trzeciak J, Lachmann B, et al (2006) A novel tool to characterize paracellular transport: the APTS-dextran ladder. Pharm Res 23: 1491–1501. 16779707 10.1007/s11095-006-0256-z

[23] Neuhaus W, Plattner VE, Wirth M, Germann B, Lachmann B, et al (2008) Validation of in vitro cell culture models of the blood-brain barrier: tightness characterization of two promising cell lines. J Pharm Sci 97: 5158–5175. 18399537 10.1002/jps.21371

[24] Neuhaus W, Stessl M, Strizsik E, Bennani-Baiti B, Wirth M, et al (2010) Blood-brain barrier cell line PBMEC/C1-2 possesses functionally active P-glycoprotein. Neurosci Lett 469: 224–228. 19963040 10.1016/j.neulet.2009.11.079

[25] Poetsch V, Neuhaus W, Noe CR (2010) Serum-derived immunoglobulins neutralize adverse effects of amyloid-beta peptide on the integrity of a blood-brain barrier in vitro model. J Alzheimers Dis 21: 303–314. 20421696 10.3233/JAD-2010-090769

[26] Neuhaus W, Lauer R, Oelzant S, Fringeli UP, Ecker GF, et al (2006) A novel flow based hollow-fiber blood-brain barrier in vitro model with immortalised cell line PBMEC/C1-2. J Biotechnol 125: 127–141. 16730091 10.1016/j.jbiotec.2006.02.019

[27] Neuhaus W, Trauner G, Gruber D, Oelzant S, Klepal W, et al (2008) Transport of a GABAA receptor modulator and its derivatives from Valeriana officinalis L. s. l. across an in vitro cell culture model of the blood-brain barrier. Planta Med 74: 1338–1344. 18704879 10.1055/s-2008-1081343

[28] Alvarez JI, Dodelet-Devillers A, Kebir H, Ifergan I, Fabre PJ, et al (2011) The Hedgehog pathway promotes blood-brain barrier integrity and CNS immune quiescence. Science 334: 1727–1731. 22144466 10.1126/science.1206936

[29] Kröll S, El-Gindi J, Thanabalasundaram G, Panpumthong P, Schrot S, et al (2009) Control of the blood-brain barrier by glucocorticoids and the cells of the neurovascular unit. Ann N Y Acad Sci 1165: 228–239. 19538311 10.1111/j.1749-6632.2009.04040.x

[30] Deli MA, Abrahám CS, Kataoka Y, Niwa M (2005) Permeability studies on in vitro blood-brain barrier models: physiology, pathology, and pharmacology. Cell Mol Neurobiol 25: 59–127. 15962509 10.1007/s10571-004-1377-8 PMC11529645

[31] Neuhaus W, Wirth M, Plattner VE, Germann B, Gabor F, et al (2008) Expression of Claudin-1, Claudin-3 and Claudin-5 in human blood-brain barrier mimicking cell line ECV304 is inducible by glioma-conditioned media. Neurosci Lett 446: 59–64. 18817843 10.1016/j.neulet.2008.09.025

[32] Schlichtiger J, Pekcec A, Bartmann H, Winter P, Fuest C, et al (2010) Celecoxib treatment restores pharmacosensitivity in a rat model of pharmacoresistant epilepsy. Br J Pharmacol 160: 1062–1071. 20590600 10.1111/j.1476-5381.2010.00765.x PMC2936016

[33] Neuhaus W, Mandikova J, Pawlowitsch R, Linz B, Bennani-Baiti B, et al (2012) Blood-brain barrier in vitro models as tools in drug discovery: assessment of the transport ranking of antihistaminic drugs. Pharmazie 67: 432–439. 22764578

[34] Pagliarulo V, Ancona P, Niso M, Colabufo NA, Contino M, et al (2013) The interaction of celecoxib with MDR transporters enhances the activity of mitomycin C in a bladder cancer cell line. Mol Cancer 12: 47. 23705854 10.1186/1476-4598-12-47 PMC3669624

[35] Lagas JS, van der Kruijssen CM, van de Wetering K, Beijnen JH, Schinkel AH (2009) Transport of diclofenac by breast cancer resistance protein (ABCG2) and stimulation of multidrug resistance protein 2 (ABCC2)-mediated drug transport by diclofenac and benzbromarone. Drug Metab Dispos 37: 129–136. 18845662 10.1124/dmd.108.023200

[36] Uchida Y, Kamiie J, Ohtsuki S, Terasaki T (2007) Multichannel liquid chromatography-tandem mass spectrometry cocktail method for comprehensive substrate characterization of multidrug resistance-associated protein 4 transporter. Pharm Res 24: 2281–2296. 17939016 10.1007/s11095-007-9453-7

[37] Tian Q, Zhang J, Chan SY, Tan TM, Duan W, et al (2006) Topotecan is a substrate for multidrug resistance associated protein 4. Curr Drug Metab 7: 105–118. 16454695 10.2174/138920006774832550

[38] El-Sheikh AA, van den Heuvel JJ, Koenderink JB, Russel FG (2007) Interaction of nonsteroidal anti-inflammatory drugs with multidrug resistance protein (MRP) 2/ABCC2- and MRP4/ABCC4-mediated methotrexate transport. J Pharmacol Exp Ther 320: 229–235. 17005917 10.1124/jpet.106.110379

[39] Yazdanian M, Briggs K, Jankovsky C, Hawi A (2004) The „High Solubility“ Definitio of the Current FDA Guidance on Biopharmaceutical Classification System May Be Too Strict for Acidic Drugs. Pharm Res. 21: 293–299. 10.1023/b:pham.0000016242.48642.71 15032311

[40] Hakala KS, Laitinen L, Kaukonen AM, Hirvonen J, Kostiainen R, et al (2003) Development of LC/MS/MS methods for cocktail dosed Caco-2 samples using atmospheric pressure photoionization and electrosprayionization. Anal Chem 75: 5969–5977. 14588039 10.1021/ac034679b

[41] Koljonen M, Hakala KS, Ahtola-Satila T, Laitinen L, Kostiainen R, et al (2006) Evaluation of cocktail approach to standardise Caco-2 permeability experiments. Eur J Pharm Biopharm 64: 379–387. 16914297 10.1016/j.ejpb.2006.06.006

[42] Smalley J, Kadiyala P, Xin B, Balimane P, Olah T (2006) Development of an on-line extraction turbulent flow chromatography tandem mass spectrometry method for cassette analysis of Caco-2 cell based bi-directional assay samples. J Chromatography B 830: 270–277. 10.1016/j.jchromb.2005.11.006 16307910

[43] Lee G, Piquette-Miller M (2003) Cytokines alter the expression and activity of the multidrug resistance transporters in human hepatoma cell lines; analysis using RT-PCR and cDNA microarrays. J Pharm Sci 92: 2152–2163. 14603501 10.1002/jps.10493

[44] Nagle MA, Wu W, Eraly SA, Nigam SK (2013) Organic anion transport pathways in antiviral handling in choroid plexus in Oat1 (Slc22a6) and Oat3 (Slc22a8) deficient tissue. Neurosci Lett 534: 133–138. 23196129 10.1016/j.neulet.2012.11.027 PMC3591490

[45] Baehr C, Reichel V, Fricker G (2006) Choroid plexus epithelial monolayers – a cell culture model from porcine brain. Cerebrospinal Fluid Res 3: 1–14. 17184532 10.1186/1743-8454-3-13 PMC1774582

[46] Nozaki Y, Kusuhara H, Kondo T, Iwaki M, Shiroyanagi Y, et al (2007) Species difference in the inhibitory effect of nonsteroidal anti-inflammatory drugs on the uptake of methotrexate by human kidney slices. J Pharmacol Exp Ther 322: 1162–1170. 17578901 10.1124/jpet.107.121491

[47] Sauer SW, Opp S, Mahringer A, Kamiński MM, Thiel C, et al (2010) Glutaric aciduria type I and methylmalonic aciduria: simulation of cerebral import and export of accumulating neurotoxic dicarboxylic acids in in vitro models of the blood-brain barrier and the choroid plexus. Biochim Biophys Acta 1802: 552–560. 20302929 10.1016/j.bbadis.2010.03.003

[48] Römermann K, Wanek T, Bankstahl M, Bankstahl JP, Fedrowitz M, et al.. (2013) (R)-[11C]verapamil is selectively transported by murine and human P-glycoprotein at the blood-brain barrier, and not by MRP1 and BCRP. Nucl Med Biol S0969-8051(13)00116-9. 10.1016/j.nucmedbio.2013.05.012 PMC3775124 23845421

[49] Angelini A, Iezzi M, Di Febbo C, Di Ilio C, Cuccurullo F, et al (2008) Reversal of P-glycoprotein-mediated multidrug resistance in human sarcoma MES-SA/Dx-5 cells by nonsteroidal anti-inflammatory drugs. Oncol Rep 20: 731–735. 18813811

[50] Awara WM, El-Sisi AE, El-Sayad ME, Goda AE (2004) The potential role of cyclooxygenase-2 inhibitors in the treatment of experimentally-induced mammary tumour: does celecoxib enhance the anti-tumour activity of doxorubicin? Pharmacol Res 50: 487–498. 15458769 10.1016/j.phrs.2004.04.002

[51] Verma N, Rettenmeier AW, Schmitz-Spanke S (2011) Recent advances in the use of Sus scrofa (pig) as a model system for proteomic studies. Proteomics 11: 776–793. 21229584 10.1002/pmic.201000320

